# Bidirectional regulation of angiogenesis and miR-18a expression by PNS in the mouse model of tumor complicated by myocardial ischemia

**DOI:** 10.1186/1472-6882-14-183

**Published:** 2014-06-05

**Authors:** Qinbo Yang, Xiaoyan Wang, Jingang Cui, Peiwei Wang, Minqi Xiong, Chenglin Jia, Li Liu, Bingbing Ning, Li Li, Wenjian Wang, Yu Chen, Teng Zhang

**Affiliations:** 1 Yueyang Hospital, Shanghai University of TCM, 110 Ganhe Rd, Shanghai 200437, China; 2 Clinical Research Institute of Integrative Medicine, Shanghai University of Traditional Chinese Medicine, Shanghai Academy of Traditional Chinese Medicine, 110 Ganhe Rd, Shanghai 200437, China

**Keywords:** Myocardial ischemia, Tumor, Angiogenesis, PNS, Bidirectional regulation, miR-18a

## Abstract

**Background:**

Panax Notoginseng Saponins (PNS) is the major class of active constituents of notoginseng, a natural product extensively used as a therapeutic agent in China. Tumor when accompanied by cardiovascular disorders poses a greater challenge for clinical management given the paradoxical involvement of angiogenesis, therefore gaining increased research attention. This study aim to investigate effects of PNS and its activity components in the mouse model of tumor complicated with myocardial ischemia.

**Methods:**

Tumor complexed with myocardial ischemia mouse model was first established, which was followed by histological and immunohistochemistry examination to assess the effect of indicated treatments on tumor, myocardial ischemia and tissue specific angiogenesis. MicroRNA (miRNA) profiling was further carried out to identify potential miRNA regulators that might mechanistically underline the therapeutic effects of PNS in this complex model.

**Results:**

PNS and its major activity components Rg1, Rb1 and R1 suppressed tumor growth and simultaneously attenuated myocardial ischemia. PNS treatment led to decreased expression of CD34 and vWF in tumor and increased expression of these vascular markers in heart. PNS treatment resulted in reduced expression of miR-18a in tumor and upregulated expression of miR-18a in heart.

**Conclusions:**

Our data demonstrated for the first time that PNS exerts tissue specific regulatory effects on angiogenesis in part through modulating the expression of miR-18a, which could be responsible for its bidirectional effect on complex disease conditions where paradoxical angiogenesis is implicated. Therefore, our study provides experimental evidence warranting evaluation of PNS and related bioactive component as a rational therapy for complex disease conditions including co-manifestation of cancer and ischemic cardiovascular disease.

## Background

Cardiovascular disease and cancer are independent leading causes of morbidity and mortality worldwide, seriously threatening human health and quality of life. With increase in the incidence of both cardiovascular disease, such as myocardial ischemia and cancer, the number of patients simultaneously laden with cardiovascular disease and malignant tumor is increased, and these complex pathological conditions pose greater challenge for clinical treatment. Moreover, anti-cancer agents or chemotherapies cause cardiovascular side effects including myocardial ischemia. Therefore, it remains as a research focus to develop therapies that present effective management of both cardiovascular disease and tumor when both conditions are coexistent in a particular patient.

It is a well-established concept that angiogenesis plays crucial roles in the pathogenesis of various diseases including tumor growth and progression and myocardial ischemia [1]. Enhanced or excessive angiogenesis is commonly observed in tumor whereas defective or insufficient angiogenesis is one of the essential pathological features implicated in myocardial ischemia. Therefore, anti-angiogenic therapy has been considered as a new modality for tumor treatment. On the other hand, treatment promoting angiogenesis is recognized as an important strategy to enhance the clinical management of myocardial ischemia [2].

Notoginseng is a one of the most extensively used medicinal herb that has a long history of clinical utilization in treating various diseases either by itself or in combination with other natural products in Traditional Chinese Medicine (TCM) [3]. The major active components of notoginseng are panax notoginseng saponins (PNS), which consist of more than 30 different types of saponins, among which ginsenoside Rg1, Rb1 are found in high content and notoginsenoside R1 a component unique to notoginseng [4]. PNS has been widely adopted as a therapeutic agent for treating cardiovascular diseases in clinic under the guidance of TCM theory [5]. Experimental evidences have been presented indicating that the cardiovascular benefits of PNS are mediated through diverse mechanisms including alleviating oxidative stress, promoting angiogenesis, modifying vasomotor function, reducing platelet adhesion, modulating ion channels, altering autonomic neurotransmitters release, and improving lipid profiles, etc [6]. Each component of PNS may exert different pharmacological effects underlined by varied mechanisms. Independent studies have shown that PNS could stimulate HUVEC proliferation, increase the numbers of invaded cells and tube branches and promote changes in the subintestinal vessels in zebrafish, supporting that PNS are equipped with proangiogenic functions [7]. Ginsenoside Rg1 has been noted as a stable proangiogenic agent in that HUVEC proliferation, migration and tube formation were significantly enhanced in the presence of Rg1 in vitro, and the density of newly formed vessels in the animals receiving Rg1 treatment witnessed significantly increase as well [8]. On the other hand, ginsenoside Rb1 exhibits inhibitory effects on proliferation and tube-like structure formation of endothelial cells in vitro [9,10]. Notoginsenoside R1 instead has been shown to be a promising compound for protecting the heart from septic shock and has anti-inflammatory effects in mice [11].

Additionally, PNS and its major components exhibit anticancer activities and have been shown to be effective against a variety of malignancies, for instance, colorectal, lung, gastric, skin, prostate and liver cancer [12]. It has been shown that PNS, Rg1, Rb1 or R1 display antiproliferative activities in tumor cells [13]. Whether or not PNS, Rg1, Rb1 or R1 exert anti-angiogenesis effects in the context of tumor growth and progression remains to be evaluated. Furthermore, whether PNS, Rg1, Rb1 or R1 could simultaneously exert proangiogenic and antiangiogenic effect when myocardial ischemia is complexed with tumor is unknown.

To address this, we established the model of lewis lung carcinoma coupled with myocardial ischemia in mouse, and investigated the effects of PNS and its major activity components Rg1, Rb1 or R1 on the tumor growth and myocardial ischemia in this complex model. We further evaluated the impact of PNS, Rg1, Rb1 or R1 on the angiogenic events and associated miR expression in tumor and heart in this complex model. Our results for the first time demonstrated a bidirectional regulatory effect of PNS on angiogenesis under complex disease conditions such as tumor coupled with myocardial ischemia, which may contribute to the simultaneous effects of PNS on suppressing tumor growth and alleviating myocardial ischemic injuries. Our data further suggest that this bidirectional effect of PNS on angiogenesis in vivo could be achieved through tissue specific modulation of the expression of proangiogenic miR-18.

## Methods

### Reagents

Panax Notoginseng Saponins (PNS) with a purity of 98% was obtained from BioAsia International Life Science Research LTD (Shanghai, China). The structures and HPLC analysis of the content of ginsenoside Rg1 (Rg1), ginsenoside Rb1 (Rb1) and notoginsenoside R1 (R1) in the PNS used for the current study was indicated in our previous publication [14]. Rg1, Rb1, and R1 with a purity of 98% were obtained from Shanghai Source Leaf Biological Technology Co. LTD (Shanghai, China). PNS, Rg1, Rb1 and R1 were dissolved in phosphate buffer solution (PBS) and stored at -20°C, and warmed up in 37°C water bath prior to each injection. Isoproterenol hydrochloride (ISO) was obtained from TOKYO Chemical Industry Co., Ltd.

### Animals

C56BL/6J male mice with approximate body weight (BW) of 20 g were purchased from Shanghai Laboratory Animal Research Center. The mice were kept under controlled laboratory conditions with temperature at 25 ± 2°C, relative humidity of 60% ± 5%,and a light-dark cycle of 12 h each. All procedures complied with the standards for the care and use of animal subjects as stated in the Guide for the Care and Use of Laboratory Animals (issued by the Ministry of Science and Technology of China, Beijing). All the procedures were reviewed and approved by Institutional Animal Care and Use Committee of Shanghai University of TCM (certificate of approval:SYXK(Hu) 2006-0001, NO.0069937).

### The complex model of lewis lung carcinoma and myocardial ischemia

Murine lewis lung carcinoma (LLC) cell line was obtained from the American Type Culture Collection (Manassas, VA, USA). The LLC cells were grown in Dulbecco’s modified Eagles medium (DMEM) supplemented with 10% fetal bovine serum. Cells were cultured at 37°C in a 5% CO2 humidified incubator. Sub-culturing was performed using standard trypsinization procedures.

To establish tumor growth in the mouse, 1 × 10^6^ LLC cells were implanted subcutaneously via a 23-gauge needle in the right inguinal region of each mouse. Solid tumors were established over a period of 10 days. After 10 days,the length and width of tumor were measured with vernier caliper. ISO was dissolved in PBS, and was delivered in the volume of 100 μL at the dose of 10 mg/kg BW through intraperitoneal injection for 10 consecutive days. All of the mice were randomly divided into 6 groups (n = 5 per group). Mice served as normal controls received PBS. Complex model was established by introducing Lewis lung carcinoma cells and administering ISO as described above. PNS treatment was further administered to the complex model at the dose of 150 mg/kg BW delivered in the volume of 100 μL via intraperitoneal injection for 20 days. Similarly, Rg1, Rb1 or R1 was administered to the complex model at the dose of 50 mg/kg BW, 50 mg/kg BW and 10 mg/kg, respectively. The choice of dosages was based upon the results from the preliminary studies, which had demonstrated an optimal effect of PNS on attenuating myocardial ischemic injuries when administered at the dose of 150 mg/kg BW. The dose of Rg1, Rb1 and R1 was calculated and estimated mainly according to the content of each component in PNS [14] and the aforementioned optimal dose of PNS. Same dose for Rg1 and Rb1 was adopted in the current study to better compare the effects of these two major saponins under complex pathological conditions. At the end of the experimental period, mice were sacrificed by overdose of anaesthetization with subcutaneous injection 100 uL 10% chloral hydrate.

### Histology and immunohistochemistry

Heart and tumor fixed in 4% paraformaldehyde were embedded in paraffin and sections were prepared in the thickness of 5 μm. Serial sections from tumor and left ventricles were collected and equally spaced sections at the interval of 10 sections were first examined by Hematoxylin & eosin (H&E) staining to analyze the pathological changes, which was followed by Masson’s trichrome staining and immunohistochemical examinations of the adjacent sections. To examine the expressions of CD34 and von Willebrand factor (vWF) in heart and tumor tissue sections, immunohistochemistry (IHC) was performed. Briefly, sections were deparaffinized in xylene and rehydrated through a graded ethanol series. After blocking with 10% normal rabbit serum in phosphate-buffered saline, the tissue sections were incubated sequentially with a rabbit anti-mouse CD34 primary antibodies (Cat#ab81289, Abcam, USA) for 30 min, followed by incubation with biotin-conjugated rat anti-rabbit IgG (Solarbio, Shanghai) for 30 min, HRP-Streptavidin (Solarbio, Shanghai) for additional 30 min and the immunoreactivity was developed using diaminobenzidine (DAB) reagent (Vector Laboratories, Burlingame, USA) for 10 min. For vWF IHC, tissue sections were first incubated with vWF primary antibodies (Cat#11778-1-AP, Solarbio, Shanghai) followed by incubation with FITC-conjugated secondary antibodies (Cat#F7367-1ML, Sigma, USA). Images were acquired using an upright Leica DM6000B microscope (Leica Microsystems, Germany).

### MicroRNA profiling analysis

Total RNA of tumor samples was isolated using mirVana™ PARIS™ kit (Cat#AM1556, Ambion, USA) following the manufacturer’s instructions. MicroRNA expression profiling was carried out using SurePrint G3 mouse v16.0 miRNA Array Kit 8×60K (Cat#G4859A, Agilent technologies, USA), which contained 1023 mouse miRNAs and 57 mouse viral miRNAs as annotated in the Sanger database miRBase16.0.

### Quantitative real time PCR analysis

After isolation of total RNA as described above, reverse transcription was carried out using miScript Reverse Transcription Kit (Qiagen, USA) and the real time PCR was performed using QuantiTect SYBR Green PCR Master Mix (Cat#218073, Qiagen, USA) following the manufacturer’s instruction on Roche LightCycler 480 II. The primers for PCR are miScript universal primer and mmu-miR-18a specific primer, 5’-TAAGGTGCATCTAGTGCAGATAG-3’. All samples were run in triplicates and normalized to the internal control U6b using primer 5’-ACGCAAATTCGTGAAGCGTT-3’. The relative expression of miRNA was calculated with 2^-[Ct(microRNA)-Ct(U6b)]^.

### Statistical analysis

Results were expressed as mean ± standard error of mean (SEM). Variance analysis was used for statistical comparisons between groups, followed by the student *t*-test using SPSS 13.0 Software. Statistical significance was set at *p* < 0.05.

## Results

### PNS or its major activity components inhibited tumor growth in the complex mouse model

**Figure 1:**
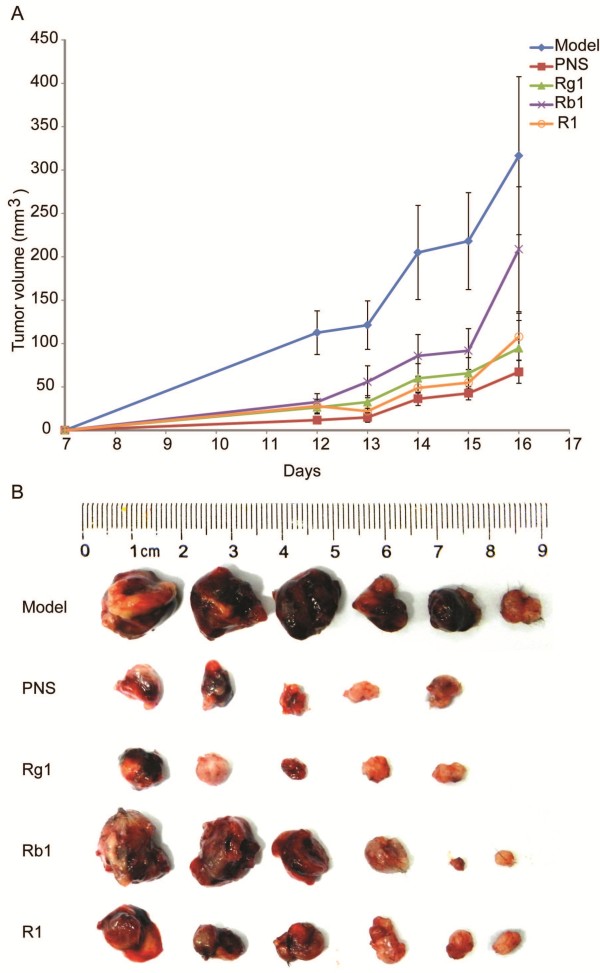
**PNS or its major active components Rg1, Rb1, and R1 inhibited tumor growth in the complex mouse model. A**. Growth curve of the tumor from vehicle-treated complex model (model), and PNS (150 mg/kg), Rg1 (50 mg/kg), Rb1 (50 mg/kg), R1 (10 mg/kg) treatment, respectively. Tumor volume (TV) (mm^3^) = (width^2^ × length)/2. **B**. Size of dissected tumor at the termination of experiment.

The complex mouse model was established by inoculating C57BL/6J mouse with LLC cells followed by isoproterenol (ISO) administration at the dose of 10 mg/kg BW for 10 days to induce myocardial ischemic injuries. ISO is a sympathomimetic β-adrenergic receptor agonist and induces infarct like cell death of cardiac muscle. The animal model of ISO-induced myocardial injury recapitulates metabolic and morphological changes occurring during human myocardial infarction, therefore, it is frequently adopted as a standardized model for evaluating the cardiac protective effects of pharmacological agents against myocardial ischemic injury [15-19]. Tumor growth of the complex mice treated with PBS vehicle or indicated treatments including PNS, Rg1, Rb1 or R1 were monitored and documented. As shown in Figure 1, tumor volume was progressively increased in the vehicle-treated complex mice. PNS, Rg1, or R1 treatment exhibited more significant effects on inhibiting tumor growth than that observed from Rb1 treatment. The tumor inhibition rates (Table 1) revealed a marked effect of PNS, Rg1, Rb1, or R1 on inhibiting tumor growth in the complex model. Note that among all the compounds examined, PNS showed the most pronounced effect on tumor inhibition in this complex mouse model.

**Table 1:** Tumor inhibition rate

**Group**	**Tumor weight (g)**	**Tumor inhibition rate (%)**
Complex model (n = 6)	0.5923 ± 0.2918	/
PNS (n = 5)	0.1037 ± 0.042	82.48%*
Rg1 (n = 5)	0.1702 ± 0.047	71.26%*
Rb1 (n = 6)	0.3536 ± 0.3395	40.30%*
R1 (n = 6)	0.2718 ± 0.3109	54.11%*

Tumor inhibition rate (%) = (1-average tumor weight in treated mice/average tumor weight in complex control) × 100%. ^*^p < 0.05 vs. complex control.

### PNS or its major activity components alleviated myocardial ischemia in the complex mouse model

**Figure 2:**
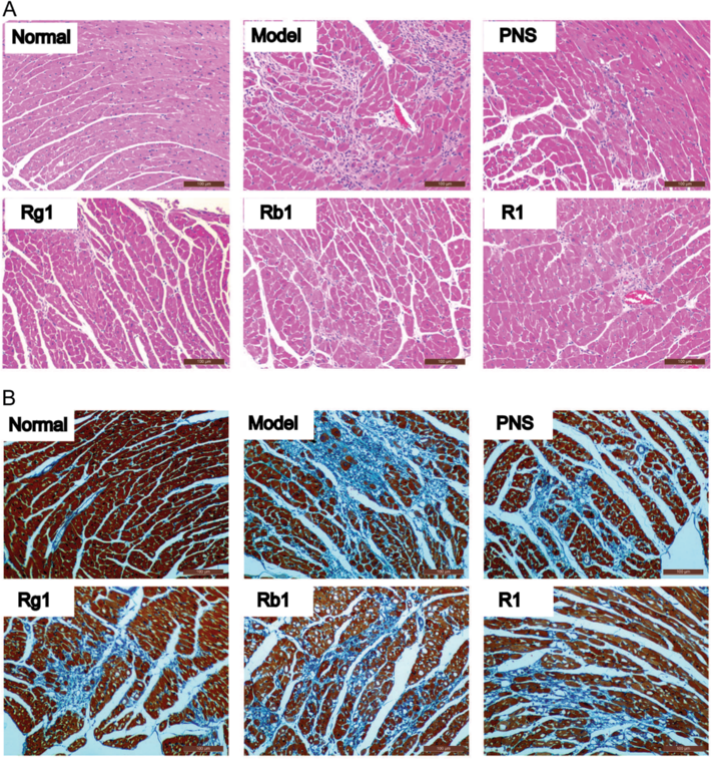
**PNS, Rg1, Rb1, and R1 treatment attenuated myocardial ischemic injuries in the complex mouse model. A**. H&E staining was performed to examine the histological changes in the hearts. Myocardial necrotic degeneration, granulation and inflammatory cell infiltration were evident in the hearts from the vehicle-treated complex mice. PNS, Rg1, Rb1, or R1 treatment significantly attenuated these pathological features in the complex mice. Scale bars indicate 100 μm. **B**. Masson’s trichrome staining was performed and revealed significant myocardial fibrosis in the vehicle-treated complex mice, which was attenuated by PNS, Rg1, Rb1, and R1 treatment, respectively. Scale bars indicate 100 μm.

Histological examination of heart was next performed in vehicle-treated normal mouse controls, vehicle-treated complex mice and complex mice receiving indicated treatment including PNS, Rg1, Rb1, or R1. As shown in Figure 2A, in normal controls, left ventricles were characterized with intact cardiac histology featuring regular organization of myocytes without any sign of necrosis. In distinct contrast, ISO induced myocardial injuries including necrotic degeneration, granulation as well as inflammatory cell infiltration, which was invariably observed in vehicle-treated complex mice. Whereas, milder necrosis and less infiltration of inflammatory cells was observed in complex mice treated with PNS, Rg1, Rb1 and R1, respectively. Myocardial ischemia associated fibrotic injury was further evaluated by Masson’s trichrome staining, a commonly adopted approach for the detection of collagen fibers in tissue sections. As shown in Figure 2B, hearts from normal controls appeared morphologically normal. However, focal fibrotic areas were readily detected in vehicle-treated complex mice, which were significantly alleviated in the complex mice treated with PNS, Rg1, Rb1, and R1, respectively (Table 2).

**Table 2:** Quantification of Masson’s trichrome staining

**Group**	**Relative area of fibrosis (%)**
Normal control	1.43 ± 0.35
Complex model	23.17 ± 8.31&
PNS	8.18 ± 3.22*
Rg1	9.64 ± 2.87*
Rb1	8.72 ± 2.13*
R1	10.16 ± 4.31*

&*p* < 0.05 vs. normal control, ^*^p < 0.05 vs. the complex model.

### PNS inhibited tumor angiogenesis, but promoted angiogenesis in ischemic heart in the complex mouse model

**Figure 3:**
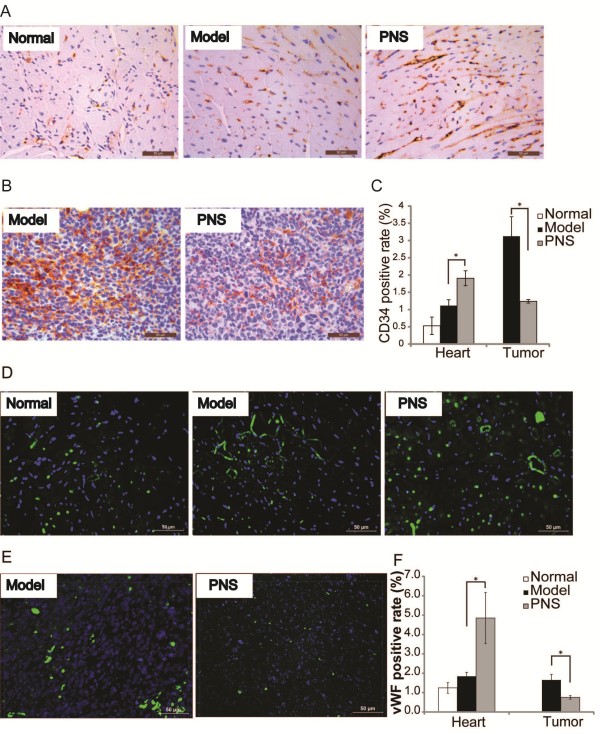
**The expression of CD34 and vWF in the heart and tumor in the complex mouse model.** Immunohistochemistry examination of heart **A**. and tumor **B**. for the expression of CD34 was examined. CD34-positive cells appeared in brown. Nuclei were stained as blue by hematoxylin. **C**. Quantification of CD34 positive staining in the heart and tumor, respectively. Immunohistochemistry examination of heart **D**. and tumor **E**. for the expression of vWF was also examined. vWF -positive cells appeared in green. Nuclei were stained as blue by DAPI. **F**. Quantification of vWF positive staining in the heart and tumor, respectively. Scale bars indicate 50 μm. *p < 0.05, compared to the vehicle-treated complex model.

Results of tumor growth and morphological examinations revealed that PNS and its major active components significantly inhibited tumor growth and meanwhile attenuated myocardial ischemic injuries in the complex mouse model. Given that angiogenesis is critically involved in tumor growth and is important for ischemic heart to recuperate, we went on to assess the impact of PNS on tumor and myocardial ischemia-associated angiogenesis in the complex mouse model. Therefore the expression of CD34 and vWF, molecular markers of vascular endothelial cell, in tumor and heart tissue sections was further evaluated by IHC. As shown in Figure 3A and C, the expressions of CD34 in heart was significantly increased in vehicle-treated complex model compared to that from the normal control, presumably as a result of ischemia-induced angiogenic response. An even greater increase in the expression of CD34 was observed in the complex model treated with PNS, suggesting a proangiogenic effect of PNS on ischemic heart. In distinct contrast, a remarkable reduction in the expression of CD34 was observed in tumor sections in PNS-treated complex mice compared to that from the vehicle-treated complex mice (Figure 3B and C), suggesting that PNS exert a significant antiangiogenic effect in growing tumor. Similar observations were made when vWF was examined to monitor the angiogenic process. As shown in Figure 3D and F, a marginal increase in vWF expression was found in the heart sections in vehicle-treated complex mice compared to that from normal controls, which was further enhanced by PNS treatment. Consistently with the result from CD34 expression analysis, a significant decrease in the expression of vWF was observed in the tumor sections of PNS-treated complex mice compared to that from the vehicle-treated complex mice (Figure 3E and F). These results demonstrated that PNS treatment led to enhanced angiogenesis in ischemic hearts whereas suppressed angiogenesis in growing tumor, implying a bidirectional regulatory action of PNS on angiogenesis in vivo depending upon the tissue type and pathological context.

### PNS regulated the expression of miR-18a in a tissue specific fashion

**Figure 4:**
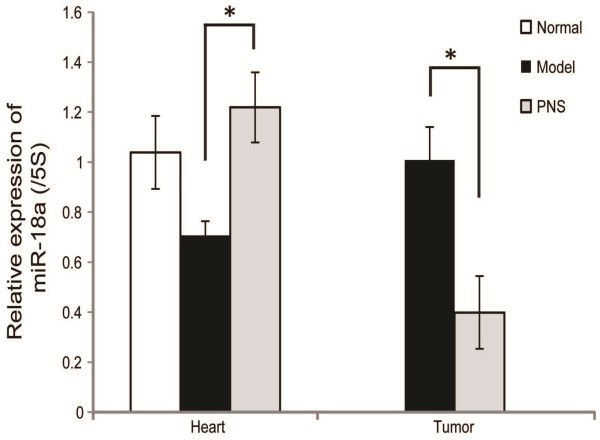
**Expression of miR-18a in heart and tumor, respectively.** Real time PCR analyses of the expression of miR-18a in heart and tumor. *p < 0.05, compared to the vehicle-treated complex model.

PNS inhibited tumor growth and attenuated the development of myocardial ischemic injuries in the complex mice, which could be attributed to its bidirectional action in modulating angiogenesis in the growing tumor and ischemic heart, respectively. To further probe the molecular mechanism that could be responsible for this bidirectional action of PNS in the process of angiogenesis, miR profiling in tumor samples was examined by microarray to uncover potential miRs affected by PNS treatment. Interesting, miR-18a, proangiogenic in its known function, was found to be differentially expressed in tumors in PNS-treated complex mice compared to that from the vehicle-treated complex mice (*p* < 0.05). Further validation of miR-18a expression was carried out by real time PCR analysis and a significant reduction in its expression in tumors from PNS-treated complex mice was confirmed compared to that from vehicle-treated controls (Figure 4). The expression of miR-18a was also examined in the hearts. The result showed that compared to that from the hearts in the vehicle-treated normal controls, the expression of miR-18a in the hearts was mildly down-regulated in the vehicle-treated complex mice, however, PNS treatment led to a significant increase in miR-18a expression compared to that from the vehicle-treated complex mice (p < 0.05). These results suggested that PNS regulated the expression of miR-18a in a tissue specific and bidirectional manner.

## Discussion

Our current study provided experimental evidence demonstrating that PNS and its major bioactive components Rg1, Rb1 or R1 exert a significant therapeutic effect on a complex disease condition featuring concomitant presentation of lewis lung carcinoma and myocardial ischemia in mouse, in which the effective therapies are confounded by paradoxical angiogenic processes in growing tumor and ischemic heart, respectively. PNS was further revealed to be able to inhibit tumor angiogenesis and in the meantime, promote myocardial ischemia-induced angiogenesis in this complex mouse model. Moreover, this bidirectional effect of PNS on tissue specific and context dependent angiogenesis is in part mediated through miR-18a expression regulation.

Therapeutic strategies targeting angiogenesis provide feasible avenues to cure a variety of diseases such as cancer and cardiovascular diseases in which aberrant angiogenesis plays a central role in the disease development and progression. The established notion that excessive angiogenesis is one of the key processes in the tumor growth and progression has led to the utilization of antiangiogenic agents in the treatment of cancer, whereas proangiogenic therapies have been explored as options to treat cardiovascular diseases given the beneficial significance of enhancing angiogenesis in post-ischemic hearts. However, neither singular antiangiogenic nor proangiogenic therapy seems to be proper for patients laden with tumor and myocardial ischemia. Moreover, therapies using monoclonal antibodies such as bevacizumab, an inhibitor of VEGF receptor, increases the survival of some patients with cancer, but also increases the risk of adverse cardiovascular effects, including hypertension, stroke and myocardial infarction [20]. In the effort of seeking rational therapies dealing with complex pathological conditions, the attention have been turned to natural products, given the fact that they most often are composed of a variety of active components that enable them to exert therapeutic effects through multiple cellular and molecular targets and in the case of PNS, may exhibit differential and bidirectional effects in a tissue and context dependent fashion [21,22].

In our current study, we identified that PNS have an ability of modulating angiogenesis with antiangiogenic activity in tumor and proangiogenic activity in ischemic heart, possibly through tissue specific regulation of the expression of miR-18a. miR-18a belongs to miR-17-92 cluster which displays potent proangiogenic activity in tumor. Studies have revealed significant overexpression of miR-18a in several types of tumor, including lung cancers, gastric cancer and oesophageal squamous cell carcinoma [23-25]. Intriguingly, a decreased expression of miR-18a and increased expression of its angiogenesis inhibitors such as connective tissue growth factor (CTGF) and thrombospondin-1 (TSP-1) were found in relation to age-related heart failure [26,27]. Therefore, the bidirectional therapeutic action of PNS in tumor complexed with myocardial ischemia model could be achieved through tissue specific regulation of the expression of miR-18a. PNS could accomplish inhibitory effect on tumor angiogenesis through downregulating the expression of miR-18a in tumor, whereas in the ischemic heart, the administration of PNS results in upregulated expression of miR-18a.

As demonstrated by the current study, when administered at the same dose, Rg1 treatment achieved a greater effect on inhibiting tumor growth compared to that from the Rb1 treatment, while similar effect of cardioprotection was observed from both Rg1 and Rb1 treatment. R1 treatment resulted in similar cardioprotection compared to that from the Rg1 and Rb1 treatment. In the meantime, R1 treatment led to greater tumor inhibition than that from the Rb1 treatment but this effect of R1 treatment was not as prominent as that from the Rg1 treatment. These results suggest a tissue specific effect of different saponin contained in PNS. Moreover, it is notable that PNS exhibited the most significant effects on tumor inhibition and cardioprotection when given at a dose that contained comparable amounts of the Rg1, Rb1 and R1. These results suggest a synergistic effect of different active components on cardioprotection and tumor inhibition, implying a better therapeutic strategy should be an integration of bioactive components at an optimal ratio, which remains to be evaluated in more details in the future studies.

The differential effects of PNS on tissue specific expression of miR-18a could be mediated by tissue specific enrichment and action of its activity components under a particular pathological condition, which remains to be further addressed. The downstream gene targets that could mediate the effects of miR-18a in response to PNS treatment in tumor and ischemic hearts requires further investigation. Therefore, the future study would be focused on analyzing miR-18a expression after administering the complex mice with each bioactive component of PNS and various combinations of bioactive components arranged at different ratios, which will shed light on the mechanisms of action of each bioactive component of PNS under complex angiogenic conditions and provide rationale for further therapeutic development treating complex disorders implicating complicated angiogenic events.

## Conclusions

Taken together, the current study demonstrated for the first time that PNS and its major activity components Rg1, Rb1 and R1 effectively suppressed tumor growth and attenuated myocardial ischemic injuries in the complex mouse model featuring simultaneous presentation of both disease conditions. These observations provide experimental evidence supporting that PNS or its major activity components Rg1, Rb1 and R1 could be considered as candidate drugs for further development to manage complex disease conditions such as cancer combined with cardiovascular disease. Furthermore, novel mechanistic evidence is presented here supporting the notion that PNS regulate angiogenesis in a bidirectional fashion through modulating the expression of miR-18a in a tissue specific and context dependent manner.

## Competing interests

The authors declare no conflict of interests.

## Authors’ contributions

QY, XW, JC, PW, MX. CJ, LL, BN, LL performed experiments and analyzed data. WW analyzed data. YC and TZ designed and experiments and analyzed the data. QY, YC and TZ wrote the manuscript. All authors read and approved the final manuscript.

## Pre-publication history

The pre-publication history for this paper can be accessed here:

http://www.biomedcentral.com/1472-6882/14/183/prepub
